# Supplementary material for: Comparative genomics provides new insights into the diversity, physiology, and sexuality of the only industrially exploited tremellomycete: Phaffia rhodozyma
Source: BMC Genomics. 2016 Nov 9;17:901. doi: 10.1186/s12864-016-3244-7 (PMC5103461; doi:10.1186/s12864-016-3244-7)
Supplement: Additional file 6: — List of orphan genes with links to PFAM (related to Additional file 1: Table S1). (ZIP 1428 kb) [file 12864_2016_3244_MOESM6_ESM.zip › BLAST_HTML_FTR/G03005_P.html]

BLAST Search Results


```
BLASTP 2.2.27+


Reference:
Stephen F. Altschul, Thomas L. Madden, Alejandro A. Schäffer,
Jinghui Zhang, Zheng Zhang, Webb Miller, and David J. Lipman (1997),
"Gapped BLAST and PSI-BLAST: a new generation of protein database
search programs", Nucleic Acids Res. 25:3389-3402.


Reference for
composition-based statistics:
Alejandro A. Schäffer, L. Aravind, Thomas L. Madden, Sergei
Shavirin, John L. Spouge, Yuri I. Wolf, Eugene V. Koonin, and
Stephen F. Altschul (2001), "Improving the accuracy of PSI-BLAST
protein database searches with composition-based statistics and
other refinements", Nucleic Acids Res. 29:2994-3005.


Database: nr
           71,551,133 sequences; 26,053,659,533 total letters


Query= G03005_P

Length=394
                                                                      Score     E
Sequences producing significant alignments:                          (Bits)  Value

emb|CED82644.1|  AT hook-like [Xanthophyllomyces dendrorhous]          786    0.0  
gb|ELT91900.1|  hypothetical protein CAPTEDRAFT_216422 [Capitella...  47.8    0.021
ref|XP_011667997.1|  PREDICTED: zinc finger protein 37-like [Stro...  47.4    0.025
gb|KFY18986.1|  hypothetical protein V493_08212 [Pseudogymnoascus...  46.6    0.039
gb|KFY94958.1|  hypothetical protein V498_03623 [Pseudogymnoascus...  46.6    0.039
gb|KFY20779.1|  hypothetical protein V491_03432 [Pseudogymnoascus...  45.4    0.081
gb|KFZ07679.1|  hypothetical protein V502_09825 [Pseudogymnoascus...  45.4    0.089
gb|KKK14348.1|  hypothetical protein ARAM_006170 [Aspergillus ram...  44.3    0.14 
ref|XP_954169.1|  Tash1-or TashAT-like protein [Theileria annulat...  44.7    0.14 
ref|XP_009035331.1|  hypothetical protein AURANDRAFT_71112 [Aureo...  45.1    0.16 
ref|WP_009349546.1|  cell division protein [Selenomonas artemidis...  44.3    0.22 
gb|KFZ16087.1|  hypothetical protein V501_02402 [Pseudogymnoascus...  43.9    0.24 
gb|KFY71081.1|  hypothetical protein V499_08733 [Pseudogymnoascus...  43.9    0.28 
gb|KJA27531.1|  hypothetical protein HYPSUDRAFT_198268 [Hypholoma...  41.2    0.34 
ref|XP_012936587.1|  PREDICTED: uncharacterized protein LOC101859...  43.1    0.60 
ref|XP_012936588.1|  PREDICTED: uncharacterized protein LOC101859...  43.1    0.62 
ref|XP_003385901.1|  PREDICTED: origin recognition complex subuni...  42.7    0.67 
gb|KFY05801.1|  hypothetical protein V492_08290, partial [Pseudog...  42.0    1.1  
ref|XP_002172747.1|  Swi5 complex subunit Swi2 [Schizosaccharomyc...  42.0    1.2  
gb|KFY43068.1|  hypothetical protein V494_02110 [Pseudogymnoascus...  42.0    1.3  
gb|KNB42673.1|  hypothetical protein JH06_3615 [Blastocystis sp. ...  41.6    1.3  
gb|EMT24783.1|  hypothetical protein F775_52518 [Aegilops tauschii]   41.6    1.8  
gb|KII88614.1|  hypothetical protein PLICRDRAFT_630327 [Plicaturo...  39.3    1.9  
ref|XP_010762841.1|  hypothetical protein PADG_07414 [Paracoccidi...  40.4    2.1  
ref|XP_012739231.1|  hypothetical protein GMDG_00837 [Pseudogymno...  41.2    2.1  
gb|EEH21734.2|  hypothetical protein PABG_03950 [Paracoccidioides...  40.4    2.2  
ref|XP_004250181.1|  PREDICTED: HMG-Y-related protein A-like [Sol...  39.3    2.5  
ref|XP_007003430.1|  hypothetical protein TREMEDRAFT_60886 [Treme...  40.4    2.6  
gb|ADD12281.1|  TashAT2 [Theileria annulata]                          40.0    2.7  
emb|CRL17977.1|  Uncharacterised protein family UPF0310 [Penicill...  40.0    3.1  
emb|CDM33744.1|  PUA-like domain [Penicillium roqueforti FM164]       40.0    3.3  
ref|XP_010551138.1|  PREDICTED: putative DNA-binding protein At1g...  39.3    3.5  
gb|KIM31764.1|  hypothetical protein M408DRAFT_64116 [Serendipita...  40.4    3.5  
ref|XP_006647291.1|  PREDICTED: uncharacterized protein LOC102715...  40.4    3.7  
ref|XP_001821215.2|  AT DNA binding protein (Thy28) [Aspergillus ...  39.7    3.7  
ref|XP_006647290.1|  PREDICTED: uncharacterized protein LOC102715...  40.4    3.9  
ref|XP_002500946.1|  predicted protein [Micromonas sp. RCC299] >g...  39.7    4.3  
gb|ETS00462.1|  hypothetical protein M419DRAFT_54688, partial [Tr...  40.0    4.5  
gb|ADD12279.1|  TashAT2 [Theileria annulata]                          38.9    5.6  
gb|KJJ25256.1|  AT hook DNA-binding motif [Penicillium solitum]       39.3    6.4  
gb|EEQ89120.1|  conserved hypothetical protein [Blastomyces derma...  39.3    6.4  
gb|KIJ16811.1|  hypothetical protein PAXINDRAFT_98610 [Paxillus i...  37.4    7.8  
gb|KIJ34672.1|  hypothetical protein M422DRAFT_51906 [Sphaerobolu...  37.7    8.5  
ref|XP_001639443.1|  predicted protein [Nematostella vectensis] >...  38.1    9.0  
gb|EIE81828.1|  hypothetical protein RO3G_06533 [Rhizopus delemar...  38.5    9.3  
gb|KKZ59016.1|  hypothetical protein EMCG_00881 [Emmonsia crescen...  38.5    9.7  


 >emb|CED82644.1| AT hook-like [Xanthophyllomyces dendrorhous]
Length=393

 Score =  786 bits (2030),  Expect = 0.0, Method: Compositional matrix adjust.
 Identities = 392/393 (99%), Positives = 392/393 (99%), Gaps = 0/393 (0%)

Query  1    MSAGSVLGLPQASSLDRSSPSSSTSSALLNRSLKWNPFPLSASDSVSPGLMSSTNVVKKR  60
            MSAGSVLGLPQASSLDRSSPSSSTSSALLNRSLKWNPFPLSASDSVSPGLMSSTNVVKKR
Sbjct  1    MSAGSVLGLPQASSLDRSSPSSSTSSALLNRSLKWNPFPLSASDSVSPGLMSSTNVVKKR  60

Query  61   GKGRPPGSKNLPKPLPGAPPPPKKKRGRPKKSVPLDPDGQPIASPPRRARGRPRRDPSSL  120
            GKGRPPGSKNLPKPLPGAPPPPKKKRGRPKKSVPL PDGQPIASPPRRARGRPRRDPSSL
Sbjct  61   GKGRPPGSKNLPKPLPGAPPPPKKKRGRPKKSVPLGPDGQPIASPPRRARGRPRRDPSSL  120

Query  121  QSPKRSRVRARPSVPVLSPVVDESGAFGVDMNINTRIMTEEIGQKEVRMTSDGSTAGWNS  180
            QSPKRSRVRARPSVPVLSPVVDESGAFGVDMNINTRIMTEEIGQKEVRMTSDGSTAGWNS
Sbjct  121  QSPKRSRVRARPSVPVLSPVVDESGAFGVDMNINTRIMTEEIGQKEVRMTSDGSTAGWNS  180

Query  181  VDIQMAVNSTGGMPTYELDSQTVKVGESGTTTGSLYQLANQPNSESIPLAQPIDSFEAFE  240
            VDIQMAVNSTGGMPTYELDSQTVKVGESGTTTGSLYQLANQPNSESIPLAQPIDSFEAFE
Sbjct  181  VDIQMAVNSTGGMPTYELDSQTVKVGESGTTTGSLYQLANQPNSESIPLAQPIDSFEAFE  240

Query  241  FPDFPFSFDFPAFLQEIQQYRRIQDDHERTGDVEGEPGHSLVSPHNPSSHPLCSSSSSST  300
            FPDFPFSFDFPAFLQEIQQYRRIQDDHERTGDVEGEPGHSLVSPHNPSSHPLCSSSSSST
Sbjct  241  FPDFPFSFDFPAFLQEIQQYRRIQDDHERTGDVEGEPGHSLVSPHNPSSHPLCSSSSSST  300

Query  301  VPPASLPSPNPLPASSHTQSLPENQTQQEQAEPTIRTRHRLSPEPPMMSEIEPIPLADSF  360
            VPPASLPSPNPLPASSHTQSLPENQTQQEQAEPTIRTRHRLSPEPPMMSEIEPIPLADSF
Sbjct  301  VPPASLPSPNPLPASSHTQSLPENQTQQEQAEPTIRTRHRLSPEPPMMSEIEPIPLADSF  360

Query  361  AFDMPAFLLEIQQYHRDHFQVKDDDAGDGDERW  393
            AFDMPAFLLEIQQYHRDHFQVKDDDAGDGDERW
Sbjct  361  AFDMPAFLLEIQQYHRDHFQVKDDDAGDGDERW  393


>gb|ELT91900.1| hypothetical protein CAPTEDRAFT_216422 [Capitella teleta]
Length=1564

 Score = 47.8 bits (112),  Expect = 0.021, Method: Composition-based stats.
 Identities = 39/99 (39%), Positives = 47/99 (47%), Gaps = 14/99 (14%)

Query  46   VSPGLMSSTNVVKKRGKGRPPGSKNLPKPLPG---APPPPKKKRGRP------KKSVPLD  96
            VSP  M+        GK +   SK    PL       P PKKK GRP      KK V L 
Sbjct  284  VSPTKMAEMGRKAVDGKRKLTQSKLTDAPLAKKSKTEPEPKKKPGRPRKNDSPKKKVVLG  343

Query  97   PDGQPIASPPRRARGRPRRDPSSLQ-SPKRSRVRARPSV  134
             DG PI    +R RGRPR+D S ++ SP++   R  P V
Sbjct  344  EDGSPI----KRGRGRPRKDGSPVKTSPRKMSPRKTPIV  378


>ref|XP_011667997.1| PREDICTED: zinc finger protein 37-like [Strongylocentrotus purpuratus]
Length=834

 Score = 47.4 bits (111),  Expect = 0.025, Method: Compositional matrix adjust.
 Identities = 23/43 (53%), Positives = 31/43 (72%), Gaps = 1/43 (2%)

Query  79   PPPPKKKRGRPKKSVPLDPDGQPIASPPRRARGRPRRDPSSLQ  121
            P P KKKRGRP+KSVP++P  Q +    +R RGRPR+  +SL+
Sbjct  168  PLPEKKKRGRPRKSVPINPP-QEVQGSAKRKRGRPRKSDTSLE  209


>gb|KFY18986.1| hypothetical protein V493_08212 [Pseudogymnoascus pannorum VKM 
F-4281 (FW-2241)]
Length=597

 Score = 46.6 bits (109),  Expect = 0.039, Method: Compositional matrix adjust.
 Identities = 31/81 (38%), Positives = 41/81 (51%), Gaps = 10/81 (12%)

Query  39   PLSASDSVSPGLMSSTNVVKKRGKGRPPGSKNLPKPLPGAPPPPKKKRGRPKKSVPLDPD  98
            PL+ +++V+P          KRG+GRP GSKNLPKP    P P K  +G+   + P  P 
Sbjct  74   PLAIAEAVTP----------KRGRGRPKGSKNLPKPDGATPTPKKLPKGKTLFATPSKPL  123

Query  99   GQPIASPPRRARGRPRRDPSS  119
            G    S  R A  R  R  S+
Sbjct  124  GLVTPSKARNAADRSARRKST  144


>gb|KFY94958.1| hypothetical protein V498_03623 [Pseudogymnoascus pannorum VKM 
F-4517 (FW-2822)]
Length=597

 Score = 46.6 bits (109),  Expect = 0.039, Method: Compositional matrix adjust.
 Identities = 29/64 (45%), Positives = 33/64 (52%), Gaps = 0/64 (0%)

Query  56   VVKKRGKGRPPGSKNLPKPLPGAPPPPKKKRGRPKKSVPLDPDGQPIASPPRRARGRPRR  115
            V  KRG+GRP GSKNLPKP    P P K  +G+   S P  P G    S  R A  R  R
Sbjct  81   VTPKRGRGRPKGSKNLPKPDGATPTPKKLPKGKTLFSTPAKPLGLLTPSKARNAADRSAR  140

Query  116  DPSS  119
              S+
Sbjct  141  RKST  144


>gb|KFY20779.1| hypothetical protein V491_03432 [Pseudogymnoascus pannorum VKM 
F-3775]
Length=598

 Score = 45.4 bits (106),  Expect = 0.081, Method: Compositional matrix adjust.
 Identities = 31/80 (39%), Positives = 40/80 (50%), Gaps = 1/80 (1%)

Query  41   SASDSVSPGLMSSTNVVK-KRGKGRPPGSKNLPKPLPGAPPPPKKKRGRPKKSVPLDPDG  99
            S  D+++ G +     V  KRG+GRP GSKNLPKP    P P K  +G+   + P  P G
Sbjct  65   SFEDAITHGSLPVVEAVTPKRGRGRPKGSKNLPKPDGATPTPKKLPKGKNLFATPSKPLG  124

Query  100  QPIASPPRRARGRPRRDPSS  119
                S  R A  R  R  S+
Sbjct  125  LLTPSKARNAADRSARRKST  144


>gb|KFZ07679.1| hypothetical protein V502_09825 [Pseudogymnoascus pannorum VKM 
F-4520 (FW-2644)]
Length=597

 Score = 45.4 bits (106),  Expect = 0.089, Method: Compositional matrix adjust.
 Identities = 28/64 (44%), Positives = 32/64 (50%), Gaps = 0/64 (0%)

Query  56   VVKKRGKGRPPGSKNLPKPLPGAPPPPKKKRGRPKKSVPLDPDGQPIASPPRRARGRPRR  115
            V  KRG+GRP GSKN PKP    P P K  +G+   S P  P G    S  R A  R  R
Sbjct  81   VTPKRGRGRPKGSKNFPKPDGATPTPKKLPKGKTLFSTPAKPLGLVTPSKARNAADRSAR  140

Query  116  DPSS  119
              S+
Sbjct  141  RKST  144


>gb|KKK14348.1| hypothetical protein ARAM_006170 [Aspergillus rambellii]
Length=331

 Score = 44.3 bits (103),  Expect = 0.14, Method: Compositional matrix adjust.
 Identities = 24/46 (52%), Positives = 31/46 (67%), Gaps = 10/46 (22%)

Query  83   KKKRGRPKK----SVPLDPDGQPIASPPRRARGRPRRDPSSLQSPK  124
            K+KRGRP+K    S P  PDG      P+R RGRPR+DPS++ +PK
Sbjct  44   KRKRGRPRKYPEGSTPKRPDG------PKRGRGRPRKDPSAIDTPK  83


>ref|XP_954169.1| Tash1-or TashAT-like protein [Theileria annulata]
 emb|CAI73492.1| Tash1-or TashAT-like protein, putative [Theileria annulata]
Length=390

 Score = 44.7 bits (104),  Expect = 0.14, Method: Compositional matrix adjust.
 Identities = 26/59 (44%), Positives = 33/59 (56%), Gaps = 8/59 (14%)

Query  80   PPPKKKRGRPKKSVPLDPD-------GQPIASPPRRARGRPRRDPSSLQSPKRSRVRAR  131
            P PKKK GRP+K  P +P+        Q   + P+R RGRPR+  S  + PKR R R R
Sbjct  136  PKPKKKLGRPRKQKP-EPETDHSEVHKQEKVAKPKRKRGRPRKQKSDREEPKRKRGRPR  193


>ref|XP_009035331.1| hypothetical protein AURANDRAFT_71112 [Aureococcus anophagefferens]
 gb|EGB10547.1| hypothetical protein AURANDRAFT_71112 [Aureococcus anophagefferens]
Length=2166

 Score = 45.1 bits (105),  Expect = 0.16, Method: Compositional matrix adjust.
 Identities = 31/59 (53%), Positives = 38/59 (64%), Gaps = 6/59 (10%)

Query  59    KRGKGRPPGSKNLPKPLPGAPPPPKKKRGRPKKSVPLDPDGQPIASPPRRARGRPRRDP  117
             K+ +GRP   +  P P  G PP PKK RGRP+K  PL  DGQP A  P++ RGRPR+ P
Sbjct  2025  KKPRGRP---RKQPPPEDGEPPAPKKPRGRPRKH-PLPEDGQPPA--PKKPRGRPRKHP  2077


>ref|WP_009349546.1| cell division protein [Selenomonas artemidis]
 gb|EFW29987.1| cell division protein FtsA [Selenomonas artemidis F0399]
Length=863

 Score = 44.3 bits (103),  Expect = 0.22, Method: Compositional matrix adjust.
 Identities = 26/64 (41%), Positives = 40/64 (63%), Gaps = 8/64 (13%)

Query  66   PGSKNLPKPLPGAPPPPKKKRGRPKKSVPLDPDGQPIASPPRRARGRPRRDPS--SLQSP  123
            P  +N+ + +P AP    K+RGRPKK +  +P+GQ     P + RGRP++D S  +L +P
Sbjct  2    PDLENINEEVP-APEQSAKRRGRPKKRIQEEPEGQ-----PAKRRGRPKKDESAPALGTP  55

Query  124  KRSR  127
            +R R
Sbjct  56   RRER  59


>gb|KFZ16087.1| hypothetical protein V501_02402 [Pseudogymnoascus pannorum VKM 
F-4519 (FW-2642)]
Length=593

 Score = 43.9 bits (102),  Expect = 0.24, Method: Compositional matrix adjust.
 Identities = 27/64 (42%), Positives = 32/64 (50%), Gaps = 0/64 (0%)

Query  56   VVKKRGKGRPPGSKNLPKPLPGAPPPPKKKRGRPKKSVPLDPDGQPIASPPRRARGRPRR  115
            V  KRG+GRP GSKN PKP    P P K  +G+   + P  P G    S  R A  R  R
Sbjct  77   VTPKRGRGRPKGSKNFPKPDGATPTPKKLPKGKTLFATPSKPLGLLTPSKARNAADRSAR  136

Query  116  DPSS  119
              S+
Sbjct  137  RKST  140


>gb|KFY71081.1| hypothetical protein V499_08733 [Pseudogymnoascus pannorum VKM 
F-103]
Length=593

 Score = 43.9 bits (102),  Expect = 0.28, Method: Compositional matrix adjust.
 Identities = 27/64 (42%), Positives = 32/64 (50%), Gaps = 0/64 (0%)

Query  56   VVKKRGKGRPPGSKNLPKPLPGAPPPPKKKRGRPKKSVPLDPDGQPIASPPRRARGRPRR  115
            V  KRG+GRP GSKN PKP    P P K  +G+   + P  P G    S  R A  R  R
Sbjct  77   VTPKRGRGRPKGSKNFPKPDGATPTPKKLPKGKTLFATPSKPLGLLTPSKARNAADRSAR  136

Query  116  DPSS  119
              S+
Sbjct  137  RKST  140


>gb|KJA27531.1| hypothetical protein HYPSUDRAFT_198268 [Hypholoma sublateritium 
FD-334 SS-4]
Length=137

 Score = 41.2 bits (95),  Expect = 0.34, Method: Compositional matrix adjust.
 Identities = 24/50 (48%), Positives = 29/50 (58%), Gaps = 5/50 (10%)

Query  59   KRGKGRPPGSKN-----LPKPLPGAPPPPKKKRGRPKKSVPLDPDGQPIA  103
            KRG+GRP GSKN     +  P P +P  P KKRGRP K    D   +P+A
Sbjct  42   KRGRGRPKGSKNKKGAAVSAPAPDSPTTPAKKRGRPPKEKKEDASEEPVA  91


>ref|XP_012936587.1| PREDICTED: uncharacterized protein LOC101859236 isoform X1 [Aplysia 
californica]
Length=2736

 Score = 43.1 bits (100),  Expect = 0.60, Method: Compositional matrix adjust.
 Identities = 35/102 (34%), Positives = 49/102 (48%), Gaps = 13/102 (13%)

Query  50    LMSSTNVVKKRGKGRPPGSKNLPKPLPGAPPP--PKKKRGRPKKSVPLDPD-----GQPI  102
             L S   V+KK   G   G +N+   +PG P P  P  KRGR +K V  + D       P+
Sbjct  1722  LSSDVKVIKKSDTGE--GEENI---IPGTPEPKSPAVKRGR-RKGVVKEKDETGSAAAPV  1775

Query  103   ASPPRRARGRPRRDPSSLQSPKRSRVRARPSVPVLSPVVDES  144
                  RARGRPR+   + +SP+ +R R          +VD+S
Sbjct  1776  VKLSVRARGRPRKAAKASESPETNRERGEEEKSFDECLVDDS  1817


>ref|XP_012936588.1| PREDICTED: uncharacterized protein LOC101859236 isoform X2 [Aplysia 
californica]
Length=2678

 Score = 43.1 bits (100),  Expect = 0.62, Method: Compositional matrix adjust.
 Identities = 35/102 (34%), Positives = 49/102 (48%), Gaps = 13/102 (13%)

Query  50    LMSSTNVVKKRGKGRPPGSKNLPKPLPGAPPP--PKKKRGRPKKSVPLDPD-----GQPI  102
             L S   V+KK   G   G +N+   +PG P P  P  KRGR +K V  + D       P+
Sbjct  1664  LSSDVKVIKKSDTGE--GEENI---IPGTPEPKSPAVKRGR-RKGVVKEKDETGSAAAPV  1717

Query  103   ASPPRRARGRPRRDPSSLQSPKRSRVRARPSVPVLSPVVDES  144
                  RARGRPR+   + +SP+ +R R          +VD+S
Sbjct  1718  VKLSVRARGRPRKAAKASESPETNRERGEEEKSFDECLVDDS  1759


>ref|XP_003385901.1| PREDICTED: origin recognition complex subunit 1-like [Amphimedon 
queenslandica]
Length=940

 Score = 42.7 bits (99),  Expect = 0.67, Method: Compositional matrix adjust.
 Identities = 38/97 (39%), Positives = 49/97 (51%), Gaps = 12/97 (12%)

Query  59   KRGKGRPPGSKNLPKP-LPGAPPPPKKKRGRPKKSVP---LDPDGQPIASPPRRARGRPR  114
            KR +GRPP +   P   L     P  +KRGRP+K V    LD   + I +P  R RGRPR
Sbjct  350  KRKRGRPPKAIKTPSDHLSEIGTPLSRKRGRPRKKVATPSLD-SSKNIVTPSSRKRGRPR  408

Query  115  RDPS--SLQSPKR-----SRVRARPSVPVLSPVVDES  144
            ++ +  SL S K      SR R RP     +P +D S
Sbjct  409  KEVATPSLDSSKNIVTPSSRKRGRPRKEEATPSLDSS  445


>gb|KFY05801.1| hypothetical protein V492_08290, partial [Pseudogymnoascus pannorum 
VKM F-4246]
Length=720

 Score = 42.0 bits (97),  Expect = 1.1, Method: Compositional matrix adjust.
 Identities = 27/64 (42%), Positives = 31/64 (48%), Gaps = 0/64 (0%)

Query  56   VVKKRGKGRPPGSKNLPKPLPGAPPPPKKKRGRPKKSVPLDPDGQPIASPPRRARGRPRR  115
            V  KRG+GRP GSKNLPKP    P P K  +G+     P    G    S  R A  R  R
Sbjct  81   VTPKRGRGRPKGSKNLPKPEGYTPTPKKLPKGKNLFGTPAKSLGLLTPSKARNAADRSAR  140

Query  116  DPSS  119
              S+
Sbjct  141  RKST  144


>ref|XP_002172747.1| Swi5 complex subunit Swi2 [Schizosaccharomyces japonicus yFS275]
 gb|EEB06454.1| Swi5 complex subunit Swi2 [Schizosaccharomyces japonicus yFS275]
Length=702

 Score = 42.0 bits (97),  Expect = 1.2, Method: Compositional matrix adjust.
 Identities = 30/75 (40%), Positives = 39/75 (52%), Gaps = 15/75 (20%)

Query  56   VVKKRGKGR-PPGSKN-----LPKPLPGAPPPPKKKRGRPKKSVPLDPDGQP-------I  102
            VVK+    R PP + N       +PLP   P PK+KRGRP+K     P G P       +
Sbjct  17   VVKQNDSNRVPPHASNENSLSTSQPLPL--PVPKRKRGRPRKQANSVPQGNPSNVKVAEL  74

Query  103  ASPPRRARGRPRRDP  117
              P +R RGRPR++P
Sbjct  75   NEPVKRKRGRPRKNP  89


>gb|KFY43068.1| hypothetical protein V494_02110 [Pseudogymnoascus pannorum VKM 
F-4513 (FW-928)]
Length=934

 Score = 42.0 bits (97),  Expect = 1.3, Method: Compositional matrix adjust.
 Identities = 27/64 (42%), Positives = 31/64 (48%), Gaps = 0/64 (0%)

Query  56   VVKKRGKGRPPGSKNLPKPLPGAPPPPKKKRGRPKKSVPLDPDGQPIASPPRRARGRPRR  115
            V  KRG+GRP GSKNLPKP    P P K  +G+     P    G    S  R A  R  R
Sbjct  81   VTPKRGRGRPKGSKNLPKPEGYTPTPKKLPKGKNLFGTPAKSLGLLTPSKARNAADRSAR  140

Query  116  DPSS  119
              S+
Sbjct  141  RKST  144


>gb|KNB42673.1| hypothetical protein JH06_3615 [Blastocystis sp. ST4]
Length=457

 Score = 41.6 bits (96),  Expect = 1.3, Method: Compositional matrix adjust.
 Identities = 25/59 (42%), Positives = 33/59 (56%), Gaps = 11/59 (19%)

Query  59   KRGKGRPPGSKNLPKPLPGAPPPPKKKRGRPKKSVPLDPDGQPIASPPRRARGRPRRDP  117
            +RG GRP       +  P   P PK+KRGRP+K    +P  +P    P+R RGRPR+ P
Sbjct  299  RRGPGRP-------RKYPVNDPLPKRKRGRPRK----NPLPEPEVPIPKRKRGRPRKHP  346


>gb|EMT24783.1| hypothetical protein F775_52518 [Aegilops tauschii]
Length=940

 Score = 41.6 bits (96),  Expect = 1.8, Method: Compositional matrix adjust.
 Identities = 34/101 (34%), Positives = 50/101 (50%), Gaps = 15/101 (15%)

Query  50   LMSSTNVVKKRGKGRPPGSKNLPKPLP--------GAPPPPKKKRGRPKKSVPLDPDGQP  101
            L+S+ + V K+G+GRP   + L   L         G    PK+ RGRP+K      D Q 
Sbjct  134  LLSANSSVTKKGRGRPRKKQKLGSSLAAFQNGTPGGGFSTPKRGRGRPRK------DAQG  187

Query  102  IASPPRRARGRPRRDPSSLQSP-KRSRVRARPSVPVLSPVV  141
            +++  +R RGRPR+D   L +  K+ R R R     +S  V
Sbjct  188  LSTRVKRGRGRPRKDAQGLSTGVKKGRGRPRKESEGMSTGV  228


>gb|KII88614.1| hypothetical protein PLICRDRAFT_630327 [Plicaturopsis crispa 
FD-325 SS-3]
Length=138

 Score = 39.3 bits (90),  Expect = 1.9, Method: Compositional matrix adjust.
 Identities = 29/62 (47%), Positives = 31/62 (50%), Gaps = 7/62 (11%)

Query  59   KRGKGRPPGSKNL-------PKPLPGAPPPPKKKRGRPKKSVPLDPDGQPIASPPRRARG  111
            KRG+GRP GSKN            P     PKKKRGRP K    D  G     PP+R RG
Sbjct  43   KRGRGRPKGSKNKKGAATSAAAAGPSTSDEPKKKRGRPPKEKKADDSGGDSEPPPKRKRG  102

Query  112  RP  113
            RP
Sbjct  103  RP  104


>ref|XP_010762841.1| hypothetical protein PADG_07414 [Paracoccidioides brasiliensis 
Pb18]
 gb|EEH42594.1| hypothetical protein PADG_07414 [Paracoccidioides brasiliensis 
Pb18]
Length=296

 Score = 40.4 bits (93),  Expect = 2.1, Method: Compositional matrix adjust.
 Identities = 27/59 (46%), Positives = 38/59 (64%), Gaps = 4/59 (7%)

Query  62   KGRPP--GSKNLPKPLPGAPPPPKKKRGRPKKSVPLDPDGQPIASPPRRARGRPRRDPS  118
            +GRPP   +   P     AP  P +KRGRP+K+ P DP  +P+A+ P+R RGRP++D S
Sbjct  14   RGRPPKLDATGAPVKKSSAPDGPPRKRGRPRKN-PNDPP-KPVATGPKRGRGRPKKDVS  70


>ref|XP_012739231.1| hypothetical protein GMDG_00837 [Pseudogymnoascus destructans 
20631-21]
 gb|ELR10425.1| hypothetical protein GMDG_00837 [Pseudogymnoascus destructans 
20631-21]
Length=594

 Score = 41.2 bits (95),  Expect = 2.1, Method: Compositional matrix adjust.
 Identities = 26/64 (41%), Positives = 31/64 (48%), Gaps = 0/64 (0%)

Query  56   VVKKRGKGRPPGSKNLPKPLPGAPPPPKKKRGRPKKSVPLDPDGQPIASPPRRARGRPRR  115
            V  K G+GRP GSKN PKP    P P K  +G+   + P  P G    S  R A  R  R
Sbjct  79   VTPKSGRGRPKGSKNFPKPDGATPTPKKLPKGKTLFATPSKPLGLLTPSKARHAADRSAR  138

Query  116  DPSS  119
              S+
Sbjct  139  RKST  142


>gb|EEH21734.2| hypothetical protein PABG_03950 [Paracoccidioides brasiliensis 
Pb03]
Length=296

 Score = 40.4 bits (93),  Expect = 2.2, Method: Compositional matrix adjust.
 Identities = 27/59 (46%), Positives = 38/59 (64%), Gaps = 4/59 (7%)

Query  62   KGRPP--GSKNLPKPLPGAPPPPKKKRGRPKKSVPLDPDGQPIASPPRRARGRPRRDPS  118
            +GRPP   +   P     AP  P +KRGRP+K+ P DP  +P+A+ P+R RGRP++D S
Sbjct  14   RGRPPKLDATGAPVKKSSAPDGPPRKRGRPRKN-PNDPP-KPVATGPKRGRGRPKKDVS  70


>ref|XP_004250181.1| PREDICTED: HMG-Y-related protein A-like [Solanum lycopersicum]
Length=160

 Score = 39.3 bits (90),  Expect = 2.5, Method: Compositional matrix adjust.
 Identities = 22/44 (50%), Positives = 30/44 (68%), Gaps = 2/44 (5%)

Query  76   PGAPPPPKKKRGRPKKSVPLDPDGQPIASPPRRARGRPRRDPSS  119
            PG   PPK+ RGRP K  P+ P G  IA+P  + RGRP++DP++
Sbjct  77   PGPDAPPKRGRGRPPKPKPILPQGTDIAAP--KPRGRPKKDPNA  118


>ref|XP_007003430.1| hypothetical protein TREMEDRAFT_60886 [Tremella mesenterica DSM 
1558]
 gb|EIW70389.1| hypothetical protein TREMEDRAFT_60886 [Tremella mesenterica DSM 
1558]
Length=362

 Score = 40.4 bits (93),  Expect = 2.6, Method: Compositional matrix adjust.
 Identities = 35/87 (40%), Positives = 43/87 (49%), Gaps = 10/87 (11%)

Query  44   DSVSPGLMSSTNVVKKRGKGRPPGSKNLPKPLP----GAPPPPKKKRGRPKKS------V  93
            DS S      + +  KRG+GRP GS+N PKP        P PP K RGRP K+       
Sbjct  173  DSSSNAEAGPSTIPSKRGRGRPKGSRNKPKPFAEIKVKPPKPPSKPRGRPLKTRLPEDQA  232

Query  94   PLDPDGQPIASPPRRARGRPRRDPSSL  120
              +   Q  A   +RARGRPR+ P  L
Sbjct  233  EYEQRKQDKAMGIQRARGRPRKFPGYL  259


>gb|ADD12281.1| TashAT2 [Theileria annulata]
Length=276

 Score = 40.0 bits (92),  Expect = 2.7, Method: Compositional matrix adjust.
 Identities = 25/57 (44%), Positives = 30/57 (53%), Gaps = 12/57 (21%)

Query  59   KRGKGRPPGSKNLPKPLPGAPPPPKKKRGRPKKSVPLDPDGQPIASPPRRARGRPRR  115
            KR  GRP   K      P  P  PK+KRGRP+K  P  P+       P+R RGRPR+
Sbjct  180  KRKPGRPRKHK------PEQPEVPKRKRGRPRKHKPEQPEQ------PKRKRGRPRK  224


>emb|CRL17977.1| Uncharacterised protein family UPF0310 [Penicillium camemberti]
Length=359

 Score = 40.0 bits (92),  Expect = 3.1, Method: Compositional matrix adjust.
 Identities = 43/122 (35%), Positives = 62/122 (51%), Gaps = 27/122 (22%)

Query  42   ASDSVSPGLMSSTNVVKKRGKGRPPG--SKNLPKPLPGAPPPPKKKRGRPKKSVPLDPDG  99
            AS S +P + ++    +KRG+GRP    + + PKP   AP   K+ RGRP+K V    + 
Sbjct  33   ASASSAPVVDATPQTGEKRGRGRPRKYPATSTPKPASDAP---KRGRGRPRKIVS---EP  86

Query  100  QPIA---SPPRRARGRPRRD--------PSSLQSPKRSRVR--------ARPSVPVLSPV  140
             P+A   S P+R RGRPR+D        P   +   R R R        A+ + P ++PV
Sbjct  87   APVAVTPSGPKRGRGRPRKDSGAATPVTPKIKKKDGRGRPRKNPLPNGEAKTTEPKVAPV  146

Query  141  VD  142
            VD
Sbjct  147  VD  148


>emb|CDM33744.1| PUA-like domain [Penicillium roqueforti FM164]
Length=333

 Score = 40.0 bits (92),  Expect = 3.3, Method: Compositional matrix adjust.
 Identities = 28/62 (45%), Positives = 42/62 (68%), Gaps = 7/62 (11%)

Query  58   KKRGKGRPPGS--KNLPKPLPGAPPPPKKKRGRPKKSVPLDPDGQPIA-SPPRRARGRPR  114
            +KRG+GRP  +   + PKP+  AP   K+ RGRP+K V  +P+ + +A + P+R RGRPR
Sbjct  54   EKRGRGRPRKNPESSAPKPISDAP---KRGRGRPRKIVS-EPEPEAVAPTGPKRGRGRPR  109

Query  115  RD  116
            +D
Sbjct  110  KD  111


>ref|XP_010551138.1| PREDICTED: putative DNA-binding protein At1g48610 isoform X2 
[Tarenaya hassleriana]
 ref|XP_010551140.1| PREDICTED: putative DNA-binding protein At1g48610 isoform X2 
[Tarenaya hassleriana]
Length=209

 Score = 39.3 bits (90),  Expect = 3.5, Method: Compositional matrix adjust.
 Identities = 26/68 (38%), Positives = 38/68 (56%), Gaps = 3/68 (4%)

Query  51   MSSTNVVKKRGKGRPPGSK--NLPKPLPGAPPPPKKKRGRPKK-SVPLDPDGQPIASPPR  107
            ++S + V KRG+GRPP  K       +    PP K+ RGRP+K   P+     P+A   +
Sbjct  6    LNSLSAVHKRGRGRPPKRKPDFHQNDVTDEAPPAKRPRGRPRKDGAPVGSKAAPMAESKQ  65

Query  108  RARGRPRR  115
            + RGRPR+
Sbjct  66   KGRGRPRK  73


>gb|KIM31764.1| hypothetical protein M408DRAFT_64116 [Serendipita vermifera MAFF 
305830]
Length=603

 Score = 40.4 bits (93),  Expect = 3.5, Method: Compositional matrix adjust.
 Identities = 32/81 (40%), Positives = 45/81 (56%), Gaps = 8/81 (10%)

Query  40   LSASDSVSPGLMSSTNVVKKRGKGRPPGSKNLPKP---LPGAPPPPKKKRGRPKKSVPLD  96
            +SA+   +P  + + ++  KRG GRP GS N  KP    P   P PK+  GRP+K V  D
Sbjct  53   VSANAQAAPDAIINGDMPVKRGPGRPKGSTNKNKPPQLGPDGNPIPKRPVGRPRKEV--D  110

Query  97   PDGQPIASPPRRARGRPRRDP  117
            P+  P+   P+   GRPR+ P
Sbjct  111  PNA-PVK--PKNPVGRPRKHP  128


>ref|XP_006647291.1| PREDICTED: uncharacterized protein LOC102715541 isoform X2 [Oryza 
brachyantha]
Length=1983

 Score = 40.4 bits (93),  Expect = 3.7, Method: Compositional matrix adjust.
 Identities = 38/119 (32%), Positives = 57/119 (48%), Gaps = 21/119 (18%)

Query  5    SVLGLPQASSLDRSSPSSSTSSALLNRSLKWNPFPLSASDSVSPGLMSSTNVVKKRGKGR  64
            S + +P A  +DR+S    +    +N+S +     L AS   +    S++   K + +GR
Sbjct  652  SCINIP-AQKIDRTSAEVGSHFQSINQSTRTENCHLEASRETT----SASPTEKPKARGR  706

Query  65   P-----PGSKNLPKPLPGAPPPPKKK---RGRPKKSVPLDPDGQPIASPPRRARGRPRR  115
            P     PG K   +  P     P  K   RGRP+K+   DPDG+P      +ARGRPR+
Sbjct  707  PRKDAEPGGKPRARGRPNKEAEPDGKPKPRGRPRKAA--DPDGKP------KARGRPRK  757


>ref|XP_001821215.2| AT DNA binding protein (Thy28) [Aspergillus oryzae RIB40]
Length=289

 Score = 39.7 bits (91),  Expect = 3.7, Method: Compositional matrix adjust.
 Identities = 32/75 (43%), Positives = 43/75 (57%), Gaps = 10/75 (13%)

Query  57   VKKRGKGRPPGSKNLPKPLPGAPPPP----KKKRGRPKKSVPLDPDGQ-PIASP-PRRAR  110
            VKKR     P ++    P   A P P    K+KRGRP+K     P+G  P  SP P+R R
Sbjct  3    VKKRKSESVPAAEENNSPKRAAVPDPATGEKRKRGRPRKY----PEGSGPKPSPGPKRGR  58

Query  111  GRPRRDPSSLQSPKR  125
            GRPR+DPS++  P++
Sbjct  59   GRPRKDPSAIGRPRK  73


>ref|XP_006647290.1| PREDICTED: uncharacterized protein LOC102715541 isoform X1 [Oryza 
brachyantha]
Length=2008

 Score = 40.4 bits (93),  Expect = 3.9, Method: Compositional matrix adjust.
 Identities = 38/119 (32%), Positives = 57/119 (48%), Gaps = 21/119 (18%)

Query  5    SVLGLPQASSLDRSSPSSSTSSALLNRSLKWNPFPLSASDSVSPGLMSSTNVVKKRGKGR  64
            S + +P A  +DR+S    +    +N+S +     L AS   +    S++   K + +GR
Sbjct  677  SCINIP-AQKIDRTSAEVGSHFQSINQSTRTENCHLEASRETT----SASPTEKPKARGR  731

Query  65   P-----PGSKNLPKPLPGAPPPPKKK---RGRPKKSVPLDPDGQPIASPPRRARGRPRR  115
            P     PG K   +  P     P  K   RGRP+K+   DPDG+P      +ARGRPR+
Sbjct  732  PRKDAEPGGKPRARGRPNKEAEPDGKPKPRGRPRKAA--DPDGKP------KARGRPRK  782


>ref|XP_002500946.1| predicted protein [Micromonas sp. RCC299]
 gb|ACO62204.1| predicted protein [Micromonas sp. RCC299]
Length=355

 Score = 39.7 bits (91),  Expect = 4.3, Method: Compositional matrix adjust.
 Identities = 32/110 (29%), Positives = 50/110 (45%), Gaps = 7/110 (6%)

Query  81   PPKKKRGRPKKSVPLDPDGQPIASPPRRARGRPRRDPSSLQSPKRSRVRARPSVPVLS--  138
            P K+ RGRP+K  P  P   P  +P +R RGRPR+D  + +     +  A+P+  V    
Sbjct  12   PAKRGRGRPRKDAP-KPIPAPKEAPAKRGRGRPRKDAPATEKTAPEKKPAKPNAAVKRRA  70

Query  139  -PVVDESGAFGVDMNINTRIMTEEIGQKEVRMTSDGSTAGWNSVDIQMAV  187
             P V E G    D +++  I  E+  ++      + +   W    I MA 
Sbjct  71   PPAVKEVGQHSDDESLDELIAAEKAKERAPPPPPEPAVENWT---ISMAT  117


>gb|ETS00462.1| hypothetical protein M419DRAFT_54688, partial [Trichoderma reesei 
RUT C-30]
Length=814

 Score = 40.0 bits (92),  Expect = 4.5, Method: Compositional matrix adjust.
 Identities = 29/82 (35%), Positives = 46/82 (56%), Gaps = 12/82 (15%)

Query  50   LMSSTNVVK--KRGKGRPPGSKNLP-------KPLPGAPPPPKKKRGRPKKSV--PLDPD  98
            +++S +V +  KRG+GRP  + ++        + +  APPP  K+RGRP K+   PL P 
Sbjct  523  VLASADVSENTKRGRGRPSNASSVTLDSTGADEDVEAAPPPAAKRRGRPSKTTAPPLLPR  582

Query  99   GQPIASPPRRARGRPRRDPSSL  120
             Q  A+  +R RGRP + P  +
Sbjct  583  -QDGAAAGKRGRGRPAKKPVEM  603


>gb|ADD12279.1| TashAT2 [Theileria annulata]
Length=225

 Score = 38.9 bits (89),  Expect = 5.6, Method: Compositional matrix adjust.
 Identities = 33/87 (38%), Positives = 41/87 (47%), Gaps = 15/87 (17%)

Query  57   VKKRGKGRPPGSKN--------LPKPLPGAPPPPKKKRGRPKKSVPLDPDGQPIASPPRR  108
            V KR  GRP   K          P+     P  PK+KRGRP+K  P +P+       P+R
Sbjct  97   VPKRRPGRPRKQKYETKKTWLLRPRKQKSQPEQPKRKRGRPRKHKP-EPEQ------PKR  149

Query  109  ARGRPRRDPSSLQSPKRSRVRARPSVP  135
             RGRPR+     + PKR R R R   P
Sbjct  150  KRGRPRKHKPEPEQPKRKRGRPRKHKP  176


>gb|KJJ25256.1| AT hook DNA-binding motif [Penicillium solitum]
Length=379

 Score = 39.3 bits (90),  Expect = 6.4, Method: Compositional matrix adjust.
 Identities = 42/128 (33%), Positives = 58/128 (45%), Gaps = 28/128 (22%)

Query  40   LSASDSVSPGLMSSTNVV-------KKRGKGRPPGS--KNLPKPLPGAPPPPKKKRGRPK  90
            L  +  V+P   +S +V+       +KRG+GRP      + PKP   AP   K+ RGRP+
Sbjct  24   LRNTVDVTPASAASASVIDATPQTGEKRGRGRPRKYPVTSTPKPASDAP---KRGRGRPR  80

Query  91   KSVPLDPDGQPIASPPRRARGRPRRD--------PSSLQSPKRSRVRARP--------SV  134
            K V          S P+R RGRPR+D        P   +   R R R  P        + 
Sbjct  81   KIVSEPAPAAVTPSGPKRGRGRPRKDSGDATPVTPKIKKKDGRGRPRKNPLPNGETKTTE  140

Query  135  PVLSPVVD  142
            P ++PVVD
Sbjct  141  PKVTPVVD  148


>gb|EEQ89120.1| conserved hypothetical protein [Blastomyces dermatitidis ER-3]
 gb|EGE81556.1| hypothetical protein BDDG_04499 [Blastomyces dermatitidis ATCC 
18188]
 gb|EQL31720.1| hypothetical protein BDFG_05950 [Blastomyces dermatitidis ATCC 
26199]
Length=311

 Score = 39.3 bits (90),  Expect = 6.4, Method: Compositional matrix adjust.
 Identities = 61/234 (26%), Positives = 92/234 (39%), Gaps = 45/234 (19%)

Query  84   KKRGRPKKSVPLDPDGQPI------ASPPRRARGRPRRDPSSL-----QSPKRSRVRAR-  131
            +KRGRP K   LD  G P+      A PPR+ RGRPR++PS L       PKR R R R 
Sbjct  8    RKRGRPPK---LDATGNPVKKLSPPAGPPRK-RGRPRKNPSELPKPAPTGPKRGRGRPRK  63

Query  132  --PSVPVLSPVVDESGAFGVDMNINTRIMTEEIGQKEVRMTSDGSTAGWNSVDIQMAVNS  189
              P+ P   P ++      +               +  ++T+D +     S DI   V S
Sbjct  64   DDPTAPSKKPRLNSD----IPTTTTATTAAPSKRGRPRKVTTDAA-----SEDINGTVES  114

Query  190  TGGMP------TYELDSQTVKVGESGTTTGSLYQLANQPNSESIPLAQPIDSFEAFEFPD  243
            + G P       Y L+ + V+  E+        +L     SES     P+     F    
Sbjct  115  SSGFPLDKIIGIYSLECKEVE--ENWPNEAEEMELTITRTSES-----PLGLIGGFNLGI  167

Query  244  FPFSFDFPAFLQEIQQYRRIQDDHERTGDVEGEP-----GHSLVSPHNPSSHPL  292
               +  F A    ++++R +       G+V+ E      G+    PH   S P+
Sbjct  168  LEGTMLFAADKPTLKKFRAVMSKSGAAGNVDEEAEASSSGNGGDLPHATQSAPV  221


>gb|KIJ16811.1| hypothetical protein PAXINDRAFT_98610 [Paxillus involutus ATCC 
200175]
Length=140

 Score = 37.4 bits (85),  Expect = 7.8, Method: Compositional matrix adjust.
 Identities = 33/79 (42%), Positives = 39/79 (49%), Gaps = 9/79 (11%)

Query  49   GLMSSTNVVKKRGKGRPPGSKNLPK-----PLPGAPPPPKKKRGRPKKSVPLDPDGQPIA  103
            G      V  KR +GRP GS+N         +P +    KKKRGRP K     PD     
Sbjct  32   GATEPNEVAPKRRRGRPKGSRNKKSGAESTTVPSSSDGVKKKRGRPPKE--KKPDDSGAE  89

Query  104  SPP-RRARGRPRRDPSSLQ  121
             PP +R RGRPR+D S LQ
Sbjct  90   EPPAKRRRGRPRKD-SQLQ  107


>gb|KIJ34672.1| hypothetical protein M422DRAFT_51906 [Sphaerobolus stellatus 
SS14]
Length=176

 Score = 37.7 bits (86),  Expect = 8.5, Method: Compositional matrix adjust.
 Identities = 31/72 (43%), Positives = 37/72 (51%), Gaps = 16/72 (22%)

Query  59   KRGKGRPPGSKNLPKPLPG-----APPPPKKKRGRPKKSVPLDPDGQPIAS--------P  105
            KRG+GRP GSKN P+         AP   K+KRGRP K  P      P  S        P
Sbjct  41   KRGRGRPKGSKNKPREGAASSSSTAPTGEKRKRGRPPKEKPA---AAPATSETGGEDNPP  97

Query  106  PRRARGRPRRDP  117
             +R RGRPR++P
Sbjct  98   AKRPRGRPRKNP  109


>ref|XP_001639443.1| predicted protein [Nematostella vectensis]
 gb|EDO47380.1| predicted protein [Nematostella vectensis]
Length=221

 Score = 38.1 bits (87),  Expect = 9.0, Method: Compositional matrix adjust.
 Identities = 27/51 (53%), Positives = 29/51 (57%), Gaps = 12/51 (24%)

Query  59   KRGKGRPPGSKNLPKPLPGAPP----------PPKKKRGRPK--KSVPLDP  97
            KR +GRP GSKN PKP PG  P          P K KRGRPK  K+ P DP
Sbjct  116  KRKRGRPKGSKNKPKPPPGEQPESTTPQEPSDPNKPKRGRPKGSKNKPKDP  166


>gb|EIE81828.1| hypothetical protein RO3G_06533 [Rhizopus delemar RA 99-880]
Length=247

 Score = 38.5 bits (88),  Expect = 9.3, Method: Compositional matrix adjust.
 Identities = 27/72 (38%), Positives = 38/72 (53%), Gaps = 6/72 (8%)

Query  83   KKKRGRPKKSVPLDPDGQPIASPPRRARGRPRRDPSSLQ---SPKRSRVRARPSV-PVLS  138
            K+KRGRP  S+    + +PI +   R RGRPR++    +   SP   R R RP   PV+ 
Sbjct  38   KRKRGRP--SIVRTEEEKPITTIIARKRGRPRKERVERKMEPSPNEHRKRGRPRKNPVIE  95

Query  139  PVVDESGAFGVD  150
               +E  AFG +
Sbjct  96   QNAEEKTAFGRE  107


>gb|KKZ59016.1| hypothetical protein EMCG_00881 [Emmonsia crescens UAMH 3008]
Length=310

 Score = 38.5 bits (88),  Expect = 9.7, Method: Compositional matrix adjust.
 Identities = 29/67 (43%), Positives = 40/67 (60%), Gaps = 4/67 (6%)

Query  60   RGKGRPPGSKNLPKPLPGAPPP--PKKKRGRPKKSVPLDPDGQPIASPPRRARGRPRRDP  117
            R +GRPP       P+    PP  P +KRGRP+K+ P +P  +P  + P+R RGRPR+D 
Sbjct  9    RKRGRPPKLDATGTPVKKPTPPDGPPRKRGRPRKN-PSEPP-KPAPTGPKRGRGRPRKDD  66

Query  118  SSLQSPK  124
            S+  S K
Sbjct  67   STAPSKK  73


Lambda      K        H        a         alpha
   0.312    0.130    0.384    0.792     4.96 

Gapped
Lambda      K        H        a         alpha    sigma
   0.267   0.0410    0.140     1.90     42.6     43.6 

Effective search space used: 3640627020344


  Database: nr
    Posted date:  Sep 23, 2015 12:05 AM
  Number of letters in database: 26,053,659,533
  Number of sequences in database:  71,551,133


Matrix: BLOSUM62
Gap Penalties: Existence: 11, Extension: 1
Neighboring words threshold: 11
Window for multiple hits: 40
```
